# Molecular Methods for the Diagnosis of Invasive Candidiasis

**DOI:** 10.3390/jof6030101

**Published:** 2020-07-06

**Authors:** Iris Camp, Kathrin Spettel, Birgit Willinger

**Affiliations:** Division of Clinical Microbiology, Department of Laboratory Medicine, Medical University of Vienna, 1090 Vienna, Austria; iris.camp@meduniwien.ac.at (I.C.); kathrin.spettel@meduniwien.ac.at (K.S.)

**Keywords:** *Candida*, invasive, diagnosis, molecular, candidemia, T2

## Abstract

Invasive infections caused by members of the genus *Candida* are on the rise. Especially patients in intensive care units, immunocompromised patients, and those recovering from abdominal surgery are at risk for the development of candidemia or deep-seated candidiasis. Rapid initiation of appropriate antifungal therapy can increase survival rates significantly. In the past, most of these infections were caused by *C. albicans*, a species that typically is very susceptible to antifungals. However, in recent years a shift towards infections caused by non-albicans species displaying various susceptibly patterns has been observed and the prompt diagnosis of the underlying species has become an essential factor determining the therapeutic outcome. The gold standard for diagnosing invasive candidiasis is blood culture, even though its sensitivity is low and the time required for species identification usually exceeds 48 h. To overcome these issues, blood culture can be combined with other methods, and a large number of tests have been developed for this purpose. The aim of this review was to give an overview on strengths and limitations of currently available molecular methods for the diagnosis of invasive candidiasis.

## 1. Introduction

The genus *Candida* comprises a diverse group of dimorphic fungi that are commensal inhabitants of mucous membranes [1] some species, like *C. parapsilosis*, additionally can be found as colonizers on the human skin [2]. *Candida* species, therefore, are often isolated from non-sterile clinical samples, such as swabs from the gastrointestinal or urogenital tract. Even though these findings usually do not hold any pathologic value in asymptomatic immunocompetent patients, *Candida* can cause invasive infections that are marked by high mortality rates [3,4]. Especially patients on intensive care units (ICU), as well as immunosuppressed or neutropenic patients, are at higher risk for the development of an invasive candidiasis (IC) [5], and improved treatment strategies and survival rates for a number of severe diseases like hematologic malignancies have led to an ever growing pool of patients susceptible to and affected by invasive fungal diseases [6]. The population-based incidence of IC has been reported to be between 1.9 and 24 cases/100,000/per year [7,8,9,10,11,12,13,14,15,16]. Worldwide, over 250,000 cases of IC and more than 50,000 deaths per year are due to these infections [17]. IC often, but not always, goes along with candidemia, and indeed *Candida* spp. have been referred to being the fourth most common cause of nosocomial blood stream infections [18].

In most cases, IC originates from the patient’s own flora, and the risk for the development of such an infection increases with the number of body sites colonized by *Candida* [19,20,21]. Even though *C. albicans* is still the species responsible for most cases of IC, more and more invasive infections due to non-albicans species have been noted in recent years [22,23,24,25,26,27]. This is relevant as not only virulence and pathogenicity but also resistance profiles vary between species. While *C. albicans* usually is susceptible to all major groups of antifungals, *C. glabrata* can acquire resistance to azoles [28], *C. parapsilosis* and *C. guilliermondii* to echinocandins [29,30], *C. lusitaniae* may be less susceptible to amphotericin B [31], and *C. krusei* isolates are intrinsically resistant to fluconazole. Thus, the observed shift towards non-albicans species makes it more difficult to choose the appropriate empiric therapy. Another concern is the emergence of *C. auris*. This often multi-resistant pathogen was first described in Japan in 2009 [32]. Since then, several outbreaks have been reported [33,34,35].

The spectrum of clinical signs and symptoms of IC is wide and can be unspecific, but an invasive fungal disease should always be considered if the patient’s condition does not improve under antibiotic therapy, especially if colonization with *Candida* spp. has been observed in a high-risk patient. To improve the outcome of IC, the prompt initiation of an appropriate antimycotic therapy is essential [36]. Given the differences in resistance patterns, fast species-level identification is required for choosing the correct antimycotic agent when results of antimicrobial susceptibility testing are not yet available.

**Figure 1:**
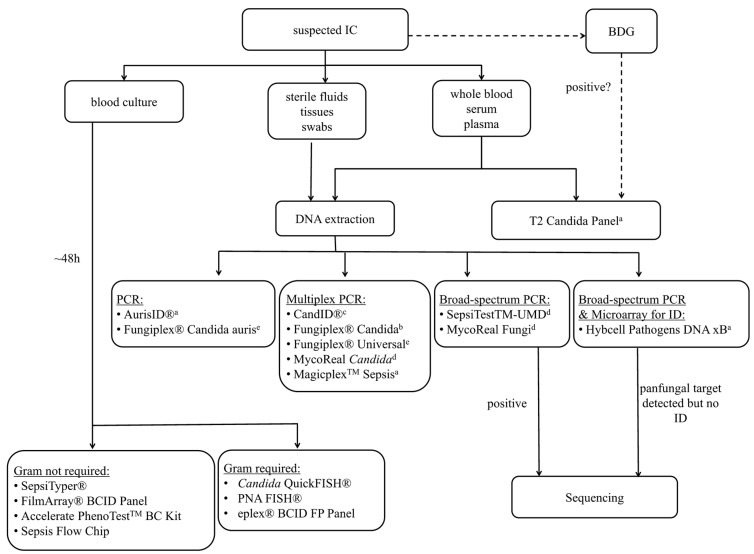
Overview of available molecular tests for the diagnosis of invasive *Candida* infections. BDG: beta-D-glucan; ^a^ whole blood, ^b^ whole blood, plasma and serum, ^c^ plasma and synthetic BAL, ^d^ various clinical samples, ^e^ extracted DNA.

Culture remains one of the key methods for diagnosing a fungal infection. However, the definitive treatment of IC is often delayed by the insensitivity of culture, and this delay may lead to high mortality rates (35–75%) [37]. Even though blood cultures (BC) are sensitive at detecting viable *Candida* cells, with a limit of detection of one colony forming unit (CFU)/mL, their overall sensitivity across the spectrum of IC is only 50%, and they have a lag time for identification of up to 5 days [38,39]. Nevertheless, BC is currently considered the “gold standard” in the event of any suspected case of invasive fungal infection, but the combination of culture with other methods can facilitate a timelier diagnosis. Molecular amplification techniques enable fast and sensitive detection and identification by directly detecting and analyzing tiny amounts of fungal DNA present in a clinical sample without the need for prior cultivation, which makes these tests appealing for the early diagnosis of IC, particularly for cases of IC that are missed by culture. Multiple PCR assays targeting various genetic sequences (18S rDNA, 28S rDNA, 5.8S rDNA, internal transcribed spacer regions and mitochondrial DNA) have been developed for the detection of a broad range of fungi in different specimens such as blood, serum, plasma, bronchoalveolar lavage (BAL), sterile fluids and tissues. Depending on the primers used (i.e., primers targeting either conserved or species-specific regions), fungal pathogens can be detected in a panfungal or a more specific manner. The sensitivity and specificity of the various techniques are variable, but mostly an improved sensitivity is observed when compared to classical cultural based methods [40]. Apart from the detection and analysis of nucleic acids, molecular assays can also be based on proteomic profiling. In this review, we will focus on commercially available tests (Figure 1).

## 2. Blood Culture-Dependent Molecular Diagnostics

These test systems (Table 1) are designed for use with aliquots of positive blood culture samples. Thus, the time required for a positive blood culture cannot be eliminated. As the pathogen load is high in positive blood culture bottles, sensitivity is not a challenge for these assays. 

The MALDI Sepsityper^®^ IVD Kit (Bruker Daltonics, Bremen, Germany) allows for pathogen identification via MALDI-TOF MS analysis from aliquots of positive blood culture bottles after a short protein extraction. In a recently published study [41], 62.5% of all *Candida* isolates could be identified with the MALDI Sepsityper^®^ IVD Kit directly from positive blood culture bottles. If a Bruker Biotyper instrument is available, this test is a rather inexpensive alternative to tests based on nucleic acid detection. Another benefit is the potential to identify all pathogens included in the database; thus, rare yeasts including *C. auris* can also be identified.

The FilmArray^®^ BCID Panel (Biomerieux, Marcy l’Etoile, France) is a Conformitè Europëenne/in vitro diagnostic (CE/IVD)-certified nested multiplex PCR system. Twenty-four pathogens, including the five most common *Candida* spp. (*C. albicans*, *C. glabrata*, *C. parapsilosis*, *C. tropicalis*, and *C. krusei*), can be detected by the assay with minimal hands-on time and a turn-around time of 1 h. In a study with both clinical and spiked samples, a sensitivity of 99.2% and a specificity of 99.9% were observed for *Candida* spp. when results of the BCID panel were compared with conventional culture [42]. In another recently published study, the FilmArray^®^ BCID panel was performed on 85 positive blood cultures with yeasts visible in the Gram stain. A total of 91 yeast strains were isolated by culture, and 84 of the isolates belonged to one of the five *Candida* species contained in the panel. All of those were identified. Seven isolates belonged to species not targeted by the test; those were not detected. Ten blood cultures contained more than one pathogen, and all pathogens included in the panel were identified correctly [43]. Since the test panel includes yeasts as well as Gram-positive and Gram-negative bacteria, it is not necessary to perform a Gram-stain prior to the assay.

The Accelerate PhenoTest^TM^ BC Kit (Accelerate Diagnostics, Tuscon, AZ, USA) is another CE/IVD approved test system that allows the identification and rapid phenotypic antimicrobial susceptibility testing of several Gram-positive and Gram-negative bacteria in positive blood cultures. In addition to bacterial pathogens, the system can detect *C. albicans* and *C. glabrata*. However, rapid susceptibility testing is not available for fungi. The test was recently evaluated in a study by Burnham et al. [44]; 10 of the 125 blood cultures positive for pathogens included in the test’s panel contained *C. glabrata*, and 5 cultures contained *C. albicans*. The assay failed to detect *C. glabrata* in two of these samples, while three false positive results with *C. glabrata*, as well as one false positive *C. albicans* result, were reported. Thus, for *C. albicans*, a sensitivity of 100% and a specificity of 99.3% were reported, while the sensitivity and specificity for *C. glabrata* were 80.0% and 97.9% respectively. Five false positive results for *C. glabrata* were also reported in a study published in 2017 [45]. At the time, this unfavorable outcome was explained by the use of the older software version (v1.0), and it was discussed that software updates might resolve the issue. 

The Sepsis Flow Chip (Master Diagnostica, Granada, Spain) is a CE and IVD approved multiplex PCR test which is able to detect 22 resistance genes in addition to more than 40 pathogens including *C. albicans* from positive BC in three hours. This is achieved by the use of biotinylated primers, automated reverse hybridization to a chip membrane and subsequent immunoenzymatic detection of positive signals. In the first evaluation on clinical samples [46], six yeast-positive BC were included. Five of these contained *C. albicans*, and all were detected by the test. One BC contained *C. parapsilosis*; for this sample, the assay did not yield a positive result. Thus, for *C. albicans*, a sensitivity of 100% was reported. 

The Candida QuickFISH^®^ and the Yeast Traffic Light PNA FISH^®^ (OpGen, Gaithersburg, MA, USA) are CE/IVD certified tests using peptide nucleic acid probes for fluorescence in situ hybridization. They can be performed directly on aliquots from yeast positive blood culture bottles (i.e., when yeast cells have been observed in the Gram stain). With the Yeast Traffic Light PNA FISH^®^, identification of *C. albicans/C. parapsilosis*, *C. tropicalis*, and *C. glabrata/C. krusei* is possible within 90 min, while the Candida QuickFISH^®^ can detect *C. albicans*, *C. parapsilosis*, and *C. glabrata* within 20 min. In a large evaluation study with 216 blood culture samples [47], the Yeast Traffic Light PNA FISH^®^ yielded the correct result in 96% of cases. One isolate of *C. parapsilosis* was misidentified as *C. tropicalis*, and a false negative result was obtained for one case of *C. parapsilosis* and one case of *C. tropicalis*. Additionally, cross reactivity with *C. bracarensis, C. nivariensis, C. orthopsilosis, N. delphensis*, and *R. mucilaginosa* was observed. Drawbacks might include the limited spectrum, as well as the requirement for a fluorescence microscope.

For the ePlex^®^ BCID (GenMark DX, Carlsbad, CA, USA), a recently CE/IVD certified system, there is a choice of three panels. Thus, depending on the results of the Gram-stain performed on positive blood cultures, the respective panel will be used. The fungal pathogen (FP) panel targets 11 *Candida* spp. (*C. albicans, C. auris, C. dubliniensis, C. famata, C. glabrata, C. guilliermondii, C. kefyr, C. krusei, C. lusitaniae, C. parapsilosis*, and *C. tropicalis*) as well as *Cryptococcus gattii, Cryptococcus neoformans, Fusarium* and *Rhodotorula*, and provides results in 1.5 h. Huang et al. tested 210 positive blood cultures from patients with blood stream infections with the appropriate panel. Yeasts were only observed in the Gram stain of seven samples; six of these samples contained *Candida* spp. included in the panel, and all of those were identified by the test. One sample contained *C. inconspicua*, which is not included in the panel and thus was not identified [48].

## 3. Blood Culture Independent Molecular Diagnostics

Blood culture independent molecular assays (Table 2) can be performed directly on whole blood, serum, or plasma samples without the need to wait for positive blood cultures. Thus, the time saving potential is higher than with blood culture dependent test systems. Since the pathogen load in the blood is low, the sensitivity of these test systems can be an issue. A large number of assays is available today for the molecular diagnosis of IC; however, many are in-house assays or commercially available research-use-only tests. Broad spectrum tests, as well as targeted multiplex assays, are available. The principle of a broad spectrum/panfungal test is to amplify conserved target regions that theoretically can be found across all fungal species. For species identification, obtained amplicons have to be analyzed further. Panfungal assays generally are less sensitive than assays targeting certain pathogens with species-specific primers, but have the ability to detect all fungal pathogens, not just the most frequent ones. An issue with the use of multiplex PCR can arise from the fact that clinicians are not usually familiar with the test panels and thus could assume that a negative multiplex result is sufficient for ruling out an invasive fungal infection. Therefore, it is important to specify which pathogens are covered on the reports created by the clinical microbiology/mycology laboratory.

Some of the tests described here come with their own DNA extraction kit, while a number of different extraction kits/protocols are recommended for other tests. As fungal cells are difficult to lyse, the protocol used for the extraction of nucleic acids might have a large effect on the assay’s outcome. Therefore, assays lacking their own DNA extraction method can be more difficult to standardize.

CandID^®^ und AurisID^®^ (Olm Diagnostics, Newcastle upon Tyne, England) are two new CE/IVD certified qPCR tests that can be performed with various real-time PCR instruments. The CandID kit detects *C. albicans, C. glabrata, C. parapsilosis, C. krusei, C. dubliniensis*, and *C. tropicalis;* the AurisID^®^ kit detects *C. auris* only. Results are available within 45 min from nucleic acid extraction; no extraction protocol/kits are recommended. According to the manufacturer, both kits have been validated with fungal cultures, the CandID^®^ kit has additionally been validated with plasma and synthetic BAL samples, the AurisID^®^ kit with blood samples. To the best of our knowledge, no studies evaluating the clinical performance of the assays have been published so far.

The Fungiplex^®^ Candida IVD PCR Kit (Bruker Daltonik, Bremen, Germany) detects *C. krusei, C. glabrata* and *Candida* spp. (including: *C. albicans*, *C. parapsilosis*, *C. tropicalis*, and *C. dubliniensis*) in whole blood, plasma and serum. For DNA extraction, kits from Qiagen and Biomerieux are recommended, and the assay manual provides instrument settings for a number of different thermocyclers. In a small prospective study on ICU patients with suspected IC, the Fungiplex^®^ Candida detected eight out of eight patients with IC and reached a sensitivity of 100% and a specificity of 94.1% [49]. Bruker also offers the panfungal Fungiplex^®^ Universal RUO PCR Kit and the Fungiplex^®^ Candida Auris RUO PCR Kit for use with extracted DNA.

The Magicplex^TM^ Sepsis Real-time Test (Seegene, Seoul, South Korea) is a CE/IVD approved multiplex real time PCR detecting 90 pathogens at genus level, and 27 pathogens, including five *Candida* spp. (*C. albicans, C. tropicalis, C. parapsilosis, C. glabrata, C. krusei*), at species level within six hours from whole blood. The Seegene Blood Pathogen Kit^TM^ is used for the pre-treatment and extraction of DNA, and this step is followed by a conventional PCR (one tube for Gram-positive bacteria and resistance markers and one tube for Gram-negative bacteria and fungi) for amplicon generation. If amplicons are detected, the conventional PCR is followed by two real-time PCRs for screening and species level identification. Seegene offers software (Seegene Viewer) for the interpretation of results. Denina et al. compared the Magicplex^TM^ test to blood culture in 150 samples from 89 patients. *Candida* spp. were detected by the Magicplex^TM^ in four samples; only one of these samples was accompanied by a positive blood culture [50]. In a recently published study, 14 patients with IC were included. In nine of these patients, *Candida* was only detected by blood culture, in two patients only by the Magicplex^TM^ assay, and in three patients by both methods [51]. Importantly, the two isolates detected only by the Magiplex^TM^ assay belonged to *C. parapsilosis*, which is well known to be a colonizing species. Thus, the detection of *C. parapsilosis* has to be interpreted with caution. Moreover, the authors describe that the test’s low sensitivity makes its implementation as a routine test in clinical microbiology laboratories difficult.

The MycoReal *Candida* (Ingenetix, Vienna, Austria) is a research-use-only multiplex PCR for the detection of *C. albicans*, *C. dubliniensis*, *C. glabrata*, *C. krusei*, *C. lusitaniae*, *C. parapsilosis*, and *C. tropicalis*. In this assay, species-specific biprobes are used. The test was evaluated in a study using both spiked and clinical samples [52]. Results of the analytical and clinical evaluation showed that this assay was highly sensitive and can be used in clinical laboratories as a simple screening test for the mentioned *Candida* species. Ingenetix also offers the MycoReal Fungi, a research-use-only panfungal test targeting the internal transcribed spacer (ITS) 2 region. The assay kit contains primers, probes, and a positive control, while the reaction mix has to be provided by the user. For DNA extraction from samples (blood, sterile fluids, tissue, paraffin embedded tissue, and BAL), a modified protocol for use with the High Pure PCR Template Preparation Kit from Roche Diagnostics is recommended. The system has been validated for the LightCycler^®^ 2.0 instrument (Roche Diagnostics), and a LoD 95% of 15 CFU/PCR is reported by the manufacturer. Obtained amplicons have to be sequenced for species identification. This test was based on an in-house test [53,54].

The SepsiTest^TM^—UMD (Molzym Molecular Diagnostics, Bremen, Germany) is a system for the CE/IVD certified broad-range detection of intact bacterial and fungal pathogens with an analytical sensitivity ranging from 10 to 80 CFU/mL. In addition to whole blood samples, this test is validated for sterile fluids, tissue samples, and swabs. For pathogen enrichment and DNA extraction, Molzym offers an automated solution, in which these steps are performed, fully automated by the SelectNA^TM^ plus robot (Micro-Dx^TM^ CE IVD). A semi-automated solution (UMD-SelectNA^TM^ CE IVD) with manual pathogen enrichment, followed by automated DNA isolation on one of the following instruments—Liaison^®^ Ixt (Diasorin), Arrow^®^ (Nordiag), Seeprep12^TM^ (Seegene), or GenoXtract^®^ (Hain Lifescience)—as well as manual extraction are possible. Afterwards, 16S and 18S rRNA genes are amplified in two separate reactions. Obtained amplicons have to be sequenced, and sequences are then analyzed with the free online SepsiTest^TM^-BLAST tool. The major advantage of this broad-range test is its wide spectrum which includes fastidious organisms that are not detectable by culture. In case of a positive PCR result, the need for sequencing increases the time to gain the result. Even though several studies have evaluated the performance of the test [55,56,57,58,59], few cases of IC were included in these studies. Schreiber et al. reported one case of *C. albicans* detected in blood cultures of a patient with a negative PCR result [56], and Nieman et al. found *C. albicans* in two patients, the yeast was only detected by blood cultures in one, and only in the PCR assay in the second patient [58].

The Hybcell Pathogens DNA xB (CubeDx, St. Valentin, Austria) is a recently CE/IVD approved test for the detection of bacteria, resistance genes, and fungi. DNA is isolated from 500 mL whole blood with the GINA pathogen enrichment and DNA purification kit (CubeDx). Subsequently, four separate PCR reactions are carried out (positive control, bacterial panel: 16S rDNA, fungal panel: 28s rDNA, and panel for the resistance markers vanA, vanB, mecA, and mecC) and a fluorescent dye is incorporated into amplicons during the PCR. Upon completion of the PCR, PCR products are transferred to cylindrical microarrays—so-called hybcells—and amplicons are identified in the hyborg device by binding to immobilized probes via elongation and detection of fluorescence signals. The system can identify 1 panbacterial target, 4 bacterial genera and 28 bacterial species, as well as 1 panfungal target, 2 fungal genera, and 13 fungal species. Thus, sequencing of the PCR products is not necessary if the pathogen is included in the test panel, and results can be available within 3 h. Should species level identification yield no result in a sample positive for the panbacterial or the panfungal target, leftover PCR products can be subjected to Sanger sequencing. This test is currently under evaluation. So far, no peer-reviewed study results are available. 

The T2Candida Panel (T2Biosystems) is a CE/IVD approved test for use on the T2Dx instrument, which utilizes T2 Magnetic Resonance (T2MR). The *Candida* panel can detect three groups of *Candida* (*C. albicans/C. tropicalis, C. glabrata/C. krusei* (which also includes *S. cerevisiae* and *C. bracarensis*), and *C. parapsilosis* (which includes *C. orthopsilosis* and *C. metapsilosis*)) in EDTA blood samples with minimal hands-on-time. After in-cartridge DNA extraction, the ITS 2 region is amplified. Amplicons are detected via hybridization with specific capture probes carrying superparamagnetic particles. The resulting agglomeration of these particles induces a shift in the sample’s magnetic resonance. This method is able to detect minimal amounts of intact target cells (1CFU/mL)—but not free DNA—with a time to result of 3–5 h. Neely et al. [60] evaluated the method for the detection of *Candida* in 2013. In their study, whole blood samples were spiked with different concentrations of *Candida* spp. included in the panel; high agreement rates between the T2MR and BC (97.8% positive and 100% negative agreement) were observed. Mylonakis et al. later evaluated the test in a large prospective study with samples from 1801 patients; 250 of these samples were spiked with *Candida* spp. [61]. The overall analytical sensitivity of the T2MR was 91.6% and its specificity was 99.4%. In 31 cases, T2MR and BC did not yield the same results; 2 patients were positive in BC but not with the T2MR, while samples from 29 patients were positive with T2MR but negative in BC. Arendrup et al. recently conducted another prospective study with 126 ICU patients which were classified into groups of proven, likely, possible, or unlikely IC based on the results of BC, culture from sterile sites, colonization, a *Candida* antigen assay, and clinical findings. Compared with BC and the antigen test, the T2Candida Panel had the highest sensitivity for the detection of IC [62], even though the sensitivity was lower than observed in the 2015 Mylonakis et al. study [61]. The best sensitivity was achieved by a combination of BC and the T2Candida Panel [62]. As the test also detects non-viable cells, T2MR might be a useful tool for the monitoring of candidemia upon initiation of antifungal therapy. This was demonstrated in a study published by Mylonakis et al in 2018 [63]. Follow- up blood samples (blood cultures and whole blood) from 31 patients with candidemia were included. Thirteen patients had at least one positive T2MR result, while BC only detected the presence of *Candida* in 4 of these 13 patients. Clancy et al. [64] compared the positivity of follow- up samples from 152 patients with candidemia. During a second blood draw, samples for T2MR and a companion BC (cBC) were obtained. Samples for the T2 were frozen and analyzed in batches. In patients under antifungal therapy, the T2MR assay was more often positive than the cBC (50% vs. 21%), whereas no difference was observed in untreated patients. Invalid reports were reported in 9% of T2 samples; this might be due to the analysis of frozen samples. The test only detects intact organisms (not free DNA), and therefore should not be performed on frozen samples. Thus, studies working with frozen samples might not reflect true performance characteristics. Zurl et al. analyzed frozen samples from 32 patients with candidemia and from 22 patients with deep- seated candidiasis [65]. Samples for T2MR testing were collected at various time points ranging from 2 days before until 5 days after the index culture (*Candida* positive BC or sterile site culture). Several invalid samples/instrument errors were observed. Furthermore, eight samples which were collected concurrently with the positive index BC yielded negative T2MR results. In two cases, the T2 detected a *Candida* species different from the species found in the BC. In the group of patients with deep-seated candidiasis, the T2Candida panel gave at least one positive result in six patients (27.3%). Remarkably, all BCs collected from patients with deep-seated candidiasis remained negative. Thus, even though the percentage of positive T2 results does not seem very high, this is an interesting observation, since the diagnosis of deep-seated candidiasis is very challenging. In addition to the T2Candida Panel, T2Biosystems also offers a research-use-only panel for the detection of *C. auris* [66] in skin swab samples. In addition to the diagnostic performance the role of T2Candida as a prognostic and patient management tool should also be evaluated. Munoz et al. showed, in a prospective observational multicenter study of patients receiving definitive antifungal treatment for candidemia, that a positive T2MR was associated with a higher risk of poor outcome, while the detection of beta-D-Glucan did not correlate with the outcome. A positive T2Candida result within the first 5 days after the report of a positive BC was an independent risk factor for complicated candidemia, defined by attributable mortality or development of metastatic, deep-seated infection [67]. In another multi-center investigation, Munoz et al. showed that T2Candida performed in patients with proven candidemia may be a better marker of complicated infection than follow-up blood cultures or detection of beta-D-glucan (BDG). These results indicate that T2Candida may influence the length and type of antifungal therapy in this population and might be used in the sense of antimicrobial stewardship [68]. As a consequence, test results could be used to expedite antifungal treatment of candidemia, and reduce overall antifungal usage without a negative effect on patient outcomes. Using T2Candida in combination with cultures is likely to offer greatest value. However, antifungal therapy may influence the sensitivity of the performance of the T2Candida Panel. As described by Clancy et al. T2Candida showed limited sensitivity (36%)/negative predictive value (NPV) (80%) in the MADRID prospective observational study under the influence of empirical antifungal therapy, whereas specificity/positive predictive value (PPV) was excellent (100%), indicating a role better suited to confirming a diagnosis or persistent infection [69]. As has been noted, stratification of high-risk patients through risk-prediction modeling is essential to achieve a sufficient pre-test probability. Irrespective of the prevalence of disease, the NPV of the T2 test is >98%, but a prevalence of around 10% may be optimal, providing a PPV and NPV of approximately 82% and 99%, respectively [69,70].

## 4. Summary

Diagnosing fungal infections has always been challenging. Advances in molecular diagnostic technologies have generated a range of tests with rapid turnaround times for the diagnosis and/or screening of patients at risk for invasive fungal infections. Increasing experience with PCR assays for the direct detection of fungi in clinical specimens and available clinical validation studies have positioned these assay types well on the way to becoming routine in clinical laboratories.

Several PCR assays, including commercially available kits, have been developed for the detection of *Candida* spp. in patients with candidemia and IC. The high sensitivity makes these assays appealing tools for the early diagnosis of IC. Depending on the method used for DNA extraction, free DNA or intact pathogen cells are detected. This difference can be relevant for patients under antifungal therapy as this may specifically influence the outcome of molecular tests that detect only intact cells. On the other hand, the interpretation of positive results from assays detecting free DNA can be challenging. Thus, the position of *Candida* PCR assays in the diagnostic algorithm of IC is not easy to establish. As published data show, *Candida* PCR has a higher sensitivity than blood culture but shows the best efficacy when used in conjunction with blood cultures and/or additional tests such as the detection of BDG. In addition, the use of molecular techniques for positive blood cultures allows a more rapid identification of *Candida* spp.

As early initiation of effective antifungal therapy is associated with improved outcomes [71], it is crucial to start a targeted therapy as early as possible. Direct molecular detection or rapid identification of *Candida* spp. from blood cultures by use of molecular assays shows the potential for early administration of an optimal antifungal therapy. In addition, these assays may allow for the correct choice of length and type of antifungal therapy, and may thus be used in the sense of antimicrobial stewardship.

However, many of these assays remain under investigation as they have not been validated for diagnosing IC in multi-center studies. The choice of adopting an in-house rather than commercial assay is dependent upon costs, as well as workflow and capacity in individual laboratories [72], and results of molecular assays should always be interpreted with caution [73]. Therefore, more data from multi-center studies is needed for a final assessment of commercial assays. 

## Figures and Tables

**Table 1:** List of blood culture-dependent tests.

Product	Manufacturer	*Candida* spp. Detected	Assay Time	Method	Approval
**no Gram stain required**					
SepsiTyper^®^	Bruker Daltonics	pan-*Candida*	15–20 min	protein extraction followed by MALDI-TOF MS	CE/IVD
FilmArray^®^ BCID Panel	Biomerieux	*C. albicans*, *C. glabrata*, *C. parapsilosis*, *C. tropicalis*, *C. krusei*	60 min	Multiplex PCR	CE/IVD
Accelerate PhenoTest^TM^ BC Kit	Accelerate Diagnostics	*C. albicans*, *C. glabrata*	90 min	automated FISH	CE/IVD
Sepsis Flow Chip	Master Diagnostica	*C. albicans*	3 h	Multiplex PCR	CE/IVD
**Gram stain required**					
Candida QuickFISH^®^	OpGen	*C. albicans* *C. parapsilosis*, *C. glabrata*	20 min	FISH	CE/IVD
Yeast Traffic Light PNA FISH^®^	OpGen	*C. albicans/C. parapsilosis*, *C. tropicalis*, *C. glabrata/C. krusei*	90 min	FISH	CE/IVD
eplex^®^ BCID FP Panel	GenMark Dx	*C. albicans*, *C. auris*, *C. dubliniensis*, *C. famata*, *C. glabrata*, *C. guilliermondii*, *C. kefyr*, *C. krusei*, *C. lusitaniae*, *C. parapsilosis*, *C. tropicalis*	90 min	Multiplex PCR	CE/IVD

MALDI-TOF MS: matrix-assisted laser desorption/ionization time of flight mass spectrometry; CE/IVD: Conformitè Europëenne/in vitro diagnostic; FISH: fluorescence in situ hybridization.

**Table 2:** List of blood culture independent tests.

Product	Manufacturer	*Candida* spp. Detected	Assay Time	Method	Approval
**DNA extraction step required**					
**Single target:**					
AurisID^®^	Olm Diagnostics	*C. auris*	45 min ^a^	qPCR	CE/IVD
Fungiplex^®^ *Candida auris*	Bruker Daltonics	*C. auris*	<2 h ^a^	Real-time PCR	RUO
**Multiplex tests:**					
CandID^®^	Olm Diagnostics	*C. albicans* *C. glabrata* *C. parapsilosis* *C. krusei* *C. dubliniensis* *C. tropicalis*	45 min ^a^	Multiplex qPCR	CE/IVD
Fungiplex^®^ Candida	Bruker Daltonics	*C. krusei* *C. glabrata* *Candida* spp. (including: *C. albicans*, *C. parapsilosis*, *C. tropicalis*, *C. dubliniensis*)	<2 h ^a^	Multiplex real-time PCR	CE/IVD
Fungiplex^®^ Universal	Bruker Daltonics	*Candida* spp.	<2 h ^a^	Multiplex real-time PCR	RUO
MycoReal Candida	Ingenetix	*C. albicans*, *C. dubliniensis*, *C. glabrata*, *C. krusei*, *C. lusitaniae*, *C. parapsilosis*, *C. tropicalis*	2 h ^a^	Multiplex PCR	RUO
Magicplex^TM^ Sepsis	Seegene	*C. albicans* *C. tropicalis* *C. parapsilosis* *C. glabrata* *C. krusei*	6 h ^b^	Multiplex PCR	CE/IVD
**Broad-spectrum tests**:					
Hybcell Pathogens DNA xB	CubeDx	Microarray: *C. albicans* *C. dubliniensis* *C. parapsilosis* *C. tropicalis* *C. glabrata* + 1 panfungal target Sequencing: pan-*Candida*	6 h ^c^	Panfungal PCR (28s) & Microarray	CE/IVD
SepsiTest^TM^-UMD	Molzym Molecular Diagnostics	pan-*Candida*	24 h	Broad-spectrum PCR (18S)	CE/IVD
MycoReal Fungi	Ingenetix	pan-*Candida*	24 h	Broad-spectrum PCR (ITS2)	RUO
**Fully automated:**					
T2 Candida Panel	T2 Biosystems	*C. albicans/tropicalis* *C. glabrata/krusei* *C. parapsilosis*	3–5 h	Multiplex PCR followed by automated T2MR based detection	CE/IVD

^a^ excluding DNA extraction; ^b^ including DNA extraction; ^c^ if sequencing is not required. CE/IVD: Conformitè Europëenne/in vitro diagnostic; RUO: research use only; ITS: internal transcribed spacer 2 region.
